# Ganglioside lipids accelerate α-synuclein amyloid formation

**DOI:** 10.1016/j.bbapap.2018.07.004

**Published:** 2018-10

**Authors:** Ricardo Gaspar, Jon Pallbo, Ulrich Weininger, Sara Linse, Emma Sparr

**Affiliations:** a Departments of Physical-Chemistry, Lund University, Sweden; b Biochemistry and Structural Biology, Lund University, Sweden; c Institute of Physics, Martin-Luther-University Halle-Wittenberg, Germany

## Abstract

The deposition of α-synuclein fibrils is one hallmark of Parkinson's disease. Here, we investigate how ganglioside lipids, present in high amounts in neurons and exosomes, influence the aggregation kinetics of α-synuclein. Gangliosides, as well as, other anionic lipid species with small or large headgroups were found to induce conformational changes of α-synuclein monomers and catalyse their aggregation at mildly acidic conditions. Although the extent of this catalytic effect was slightly higher for gangliosides, the results imply that charge interactions are more important than headgroup chemistry in triggering aggregation. In support of this idea, uncharged lipids with large headgroups were not found to induce any conformational change and only weakly catalyse aggregation. Intriguingly, aggregation was also triggered by free ganglioside headgroups, while these caused no conformational change of α-synuclein monomers. Our data reveal that partially folded α-synuclein helical intermediates are not required species in triggering of α-synuclein aggregation.

## Graphical abstract

**Figure:**
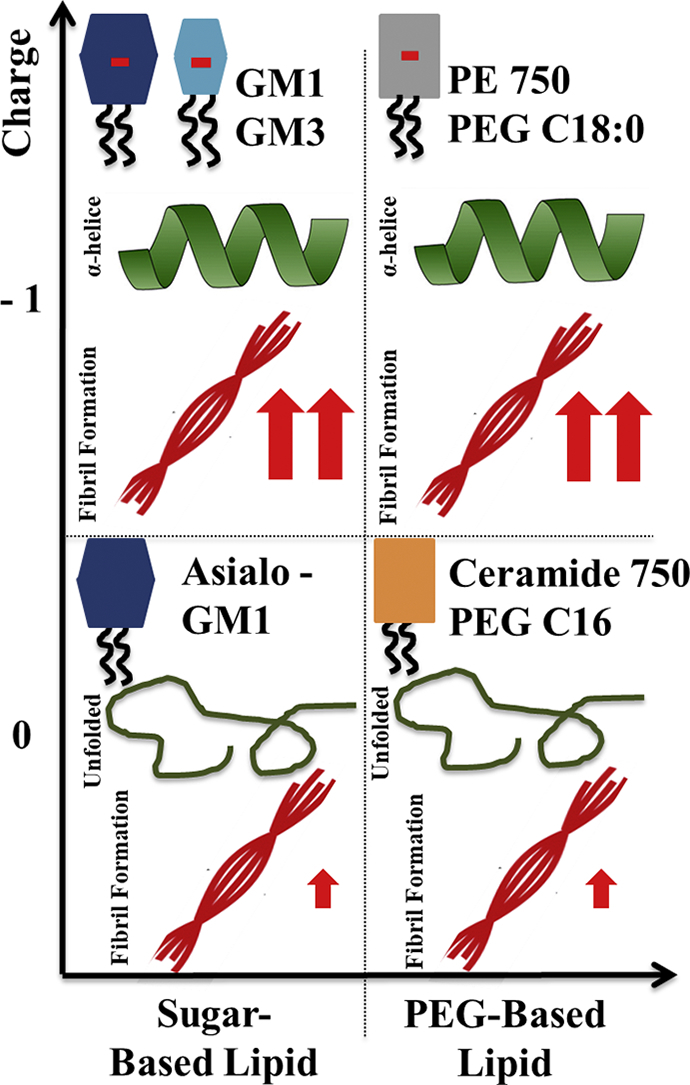

## 1 Introduction

Parkinson's disease (PD) is the second most common neurodegenerative disorder. The pathological hallmark of PD is the formation of intracellular protein aggregates, known as Lewy bodies, which are composed of α-synuclein (α-syn) amyloid fibrils [1, 2]. *In vitro*, α-syn has been shown to aggregate into β-sheet enriched amyloid fibrils with a structure resembling those found in aggregate protein deposits *in vivo* [3]. The 140-amino acid-long α-syn protein is expressed at high levels in the brain and accounts for about 1% of the total protein content in the neuronal cytosol [4]. While the normal function of α-syn *in vivo* is still not fully understood, it is believed to modulate synaptic plasticity and presynaptic vesicle pool size, of importance for neurotransmitter release, as well as, vesicle recycling [5, 6].

For several of the amyloid disorders, including PD, protein aggregation has been associated with membrane disruption in cells and in model membranes [[7], [8], [9]]. There are growing evidences from *in vivo* and *in vitro* studies that lipids are incorporated in the amyloid plaques, which can thus be viewed as lipid-protein co-aggregates [[10], [11], [12], [13], [14], [15], [16], [17], [18], [19]]. Numerous studies have reported that lipid membranes can influence the process of protein aggregation [[20], [21], [22]]. Interactions between lipids and proteins may thus govern the aggregation process, as well as, the structure and properties of the formed aggregates.

The interactions between an aggregating amyloid protein and lipid membranes will naturally depend on the molecular properties of the system, including protein structure, net charge and charge distribution, as well as, solution conditions and membrane composition and phase behavior [[23], [24], [25], [26], [27]]. In the living system, the cell membranes have complex lipid compositions, including zwitterionic and anionic lipids with varying chain lengths and headgroup chemistry. Therefore, the molecular understanding of lipid-protein interactions in amyloid formation relies on studies of simplified model systems. Model membranes can be used to dissect the role of individual components found in membrane systems *in vivo* and to study how the membrane composition affects the protein aggregation related to PD. Ganglioside lipids are one class of lipids that are primarily found in the outer plasma membrane, and they are present at relatively high concentrations (up to ca 10 wt% [28]) in neurons. Gangliosides have also been identified in cell-derived vesicles, for example, exosomes [21]. The gangliosides are anionic lipids that consist of a glycosphingolipid with one or more sialic acids (*e.g. n*-acetylneuraminic, NANA) that are linked to an oligosaccharide headgroup. These lipids have been associated with several physiological processes, including cell signaling, neuronal protection, neuronal recovery, and apoptosis [[29], [30], [31]]. Gangliosides have also been associated with the formation of nanodomains in cell membranes [32, 33], and there are several reports showing that ganglioside-containing membranes may catalyse the aggregation of the amyloid β protein (Aβ, [[34], [35], [36]]), which is associated with plaque formation in Alzheimer's disease (AD). We recently demonstrated that ganglioside-containing exosomes, as well as vesicles prepared from extracted exosome lipids and ganglioside-containing model mixtures have the effect to accelerate α-syn aggregation [21]. Secretion of α-syn *via* exosomes has been proposed to amplify and propagate PD pathology [[37], [38], [39]], and several studies have identified α-syn associated with exosomes [[40], [41], [42]]. We here address a question raised in these previous studies: are the effects observed for ganglioside-containing membranes related to specific interactions between the protein and the ganglioside headgroup, or is it attributed to more generic properties of the lipid, such as, headgroup charge and size, or lipid self-assembly?

α-Syn is an intrinsically disordered protein, *i.e.*, its monomer in aqueous solution is unfolded, while in the presence of anionic vesicles or micelles, it adopts a helical conformation [[43], [44], [45], [46]]. α-Syn can be described as consisting of three sequence domains, including the effectively positively charged N-terminus (residues 1–60), the central hydrophobic so-called NAC region involved in fibril formation (residues 61–95) and the negatively charged C-terminus (residues 96–140) [47]. When associated to anionic phospholipid membranes, the N-terminal domain adopts an α-helical conformation. The adsorbed monomeric protein is then located in the interfacial layer close to the lipid headgroups and it does not appear to penetrate deeply into the hydrophobic acyl chain region of the membrane [48, 49]. When associated to anionic phospholipid vesicles α-syn forms a linear helix [50]. The interactions between α-syn and membranes have been found to modulate the α-syn fibril formation pathway, likely through triggering heterogeneous primary nucleation [8, 20, 24, 25, [51], [52], [53], [54], [55]].

**Fig. 1:**
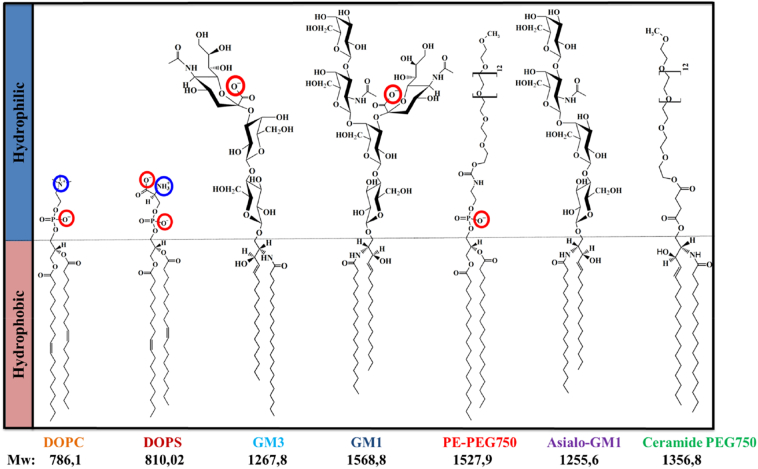
Chemical structures of all lipid species investigated: DOPC, DOPS, GM3, GM1, PE-PEG750, Asialo-GM1, and Ceramide PEG750. The same color coding of the lipid names with be used for data in Fig. 2, Fig. 3, Fig. 4, Fig. 5.

Anionic ganglioside lipids have large oligosaccharide headgroups, and thereby clearly differ from the more commonly studied anionic phospholipids with relatively small headgroups. This difference may have consequences for protein-membrane association and catalysis of nucleation due to specific interactions with the sugar moiety. This difference may also affect the entropic repulsive interactions between the vesicles and molecules in solution related to the large hydrophilic ganglioside headgroup [56]. In the present paper, we characterize the effects of ganglioside lipids on α-syn aggregation, and through comparisons with other lipid systems, we specifically target the effects of charge, size and other aspects of the lipid headgroup chemistry. We used model membranes composed of zwitterionic DOPC mixed with 10 mol% ganglioside lipids, GM1 or GM3 (Fig. 1), and we have then systematically exchanged the GM lipids with other natural or synthetic lipids, and studied how this influences α-syn secondary structure and aggregation. We also investigated the influence of free sugar species on the protein aggregation in the absence of lipid membranes. The experiments were performed at mildly acidic pH and low ionic strength (10 mM MES/NaOH buffer pH 5.5). Under these solution conditions, the dominant microscopic aggregation event for α-syn alone is secondary nucleation, which involves nucleation of monomers on the surface of existing fibrils [26, 57]. Mildly acidic pH has physiological relevance because α-syn is found in some cellular compartments, such as lysosomes and endosomes, with an acidic lumen.

## 2 Results

**Fig. 2:**
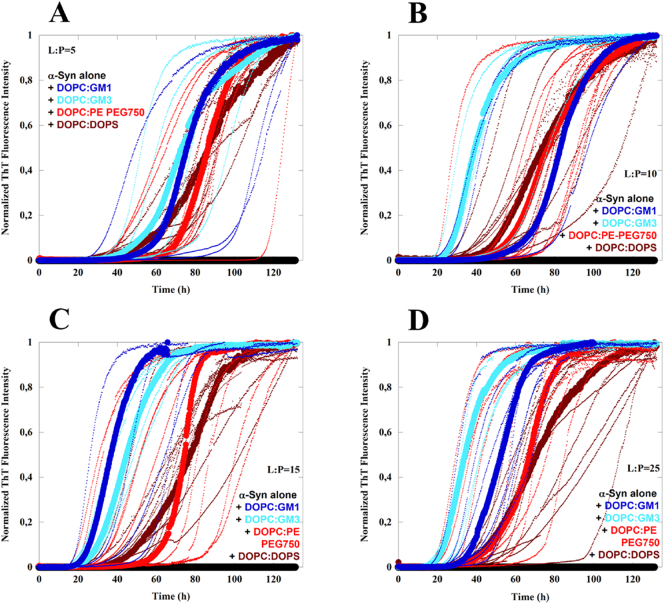
Aggregation kinetics of 20 μM α-syn in the presence of lipid vesicles in 10 mM MES pH 5.5 under quiescent conditions at 37 °C. A-D) The aggregation kinetics monitored using ThT fluorescence for α-syn alone (black traces) and in the presence of lipid vesicles composed of (molar ratios) 9:1 DOPC:GM1 (blue traces), 9:1 DOPC:GM3 (light blue traces), 9:1 DOPC:PE-PEG750 (red traces) and 9:1 DOPC:DOPS (dark red traces) at total lipid concentrations ranging from 0.1 to 0.5 mM, with the lipid-to-protein molar ratios given in each panel. The median experimental replicate for each condition is shown as a solid line with other experimental replicates dotted.

Aggregation assays under quiescent conditions, monitored by ThT fluorescence, were used to dissect the role of different molecular features of ganglioside lipids in their catalysis of α-syn aggregation. The effect of GM1 and GM3 was thus compared to other anionic lipids (Fig. 1), either a synthetic PEG-ylated phospholipid with a similar size of the hydrophilic headgroup of GM1, or a common phostadylserine, DOPS, that has a relatively small headgroup. We have also studied the effects of removing charges from large headgroups (Fig. 1), using Asialo-GM1 and a PEG-ylated ceramide and from small headgroups using DOPC. To discriminate between the role of the sugar headgroup *per se* and its presence in a lipid membrane, we studied the effects of GM3 headgroups and various sugars. α-Syn aggregation is extremely sensitive to intrinsic and extrinsic factors, and it is therefore crucial for the kinetic studies to use experimental conditions that lead to reproducible aggregation kinetics and minimal interference from heterogeneous nucleation at external surfaces that are not part of the lipid-protein system investigated [64], [65], [66]. In particular, surface material of the sample containers has a large influence on the α-syn aggregation pathway, where, for example, non-treated polystyrene (PS) plates induce aggregation [57]. Therefore, all aggregation kinetics data shown here, were performed in non-binding PEG-ylated plates. The experiments were done at slightly acidic pH (pH 5.5) in conditions where the dominant microscopic aggregation event is secondary nucleation [57]. In this environment and under quiescent conditions, α-syn in the absence of catalytic surfaces does not aggregate up to several days (~140 h) (Fig. 2A-D, black curves).

### 2.1 Ganglioside lipids *versus* other negatively charged lipids

We first investigated how the process of α-syn aggregation is affected by the presence of lipid vesicles containing different types of anionic lipids (Fig. 2). For all cases examined, aggregation is triggered and starts within 20–60 h (Fig. 2A-D). The lipid vesicles were composed of 90 mol% DOPC and 10 mol% anionic lipid, where the anionic component was either ganglioside (GM1 or GM3) or a PEG-ylated PE (PE-PEG750) (Fig. 1). As a reference system, we also studied the effect of anionic DOPS. All these lipid vesicle systems show strong accelerating effect on the aggregation of α-syn. The data in Fig. 2 also imply an effect of the lipid-to-protein ratio with shorter lag-times at higher lipid contents. At the highest lipid concentrations, the vesicles containing ganglioside lipids cause slightly stronger acceleration than vesicles containing the smaller but also negatively charged DOPS (Fig. 2C-D).

### 2.2 Lipid-induced changes in secondary structure

**Fig. 3:**
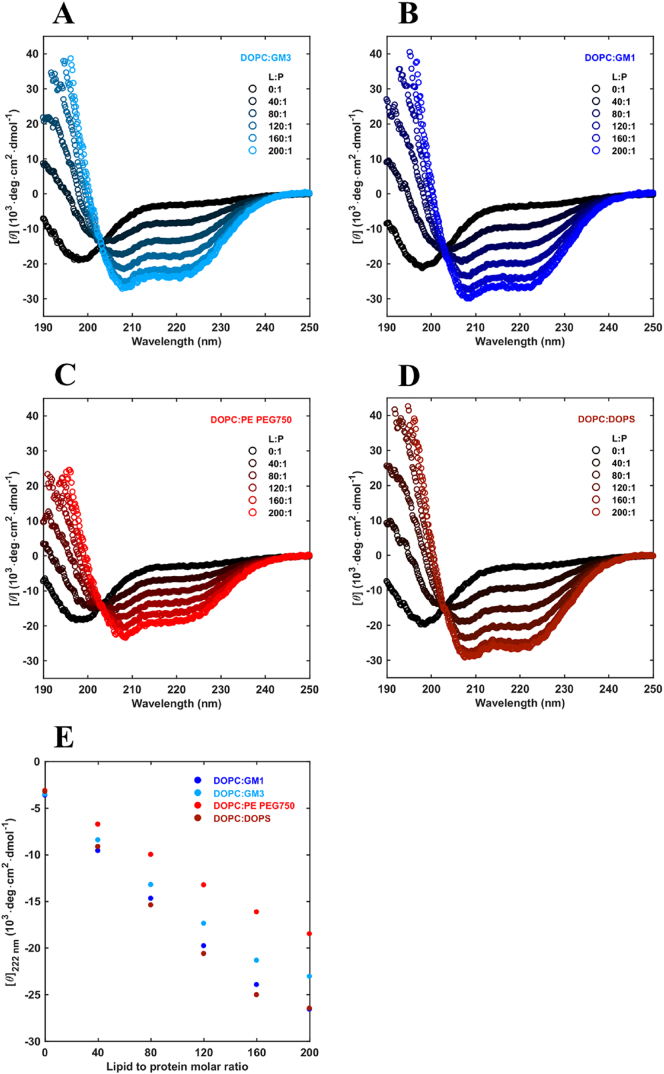
Circular Dichroism (CD) spectra. A-D) Far UV CD spectra of 5 μM α-syn alone and when incubated with 9:1 DOPC:GM3 (light blue), 9:1 DOPC:GM1 (blue), 9:1 DOPC:PE-PEG 750 (red) and 9:1 DOPC:DOPS (dark red) at different lipid:protein ratios in 10 mM MES pH 5.5 at 37 °C at total lipid concentrations ranging from 0.2 mM to 1 mM, with the lipid-to-protein molar ratios given in each panel. E) The mean residue ellipticity at wavelength 222 nm is plotted as a function of lipid:protein ratio for the 4 different lipid systems.

Changes in secondary structure of α-syn in the initial stages of the aggregation reaction were monitored using circular dichroism spectroscopy (Fig. 3A-D). In the presence of lipid membranes containing GM1, GM3, PE-PEG750 or DOPS, signature α-helical spectra were observed with two characteristic minima at wavelengths 208 nm and 222 nm. The peak intensity at 222 nm, which is correlated to the α-helical content, was plotted in Fig. 3E *versus* lipid:protein ratio ranging from 0:1 to 100:1. For all anionic vesicles that trigger aggregation (Fig. 2), a signal change indicative of a conformational change from random coil to α-helix was observed (Fig. 3). The trend is similar for all anionic lipid systems investigated, however, there is clearly a more pronounced increase in α-helical signal of α-syn in the presence of ganglioside and DOPS containing vesicles as compared to the PE-PEG750 vesicles (Fig. 3E). The observed signal is a time- and ensemble average over the whole sample; thus this could reflect either the same amount of helix per bound protein and a smaller fraction of bound protein, or the same fraction of bound protein with a smaller amount of helix per bound protein, or something in-between.

### 2.3 The importance of headgroup charge

In an attempt to further explore the importance of charge of the ganglioside lipids, we investigated systems containing uncharged lipid species with different headgroup size and chemistry. We used two uncharged lipids with large hydrophilic headgroups: a truncated GM1 lipid stripped of NANA, (Asialo-GM1) and a PEG-ylated Ceramide (Cer-PEG750). These lipids were mixed with DOPC at a molar ratio of 9:1, analogous to the studies of anionic vesicles described above. As a reference system, we also studied the effect of vesicles containing only DOPC, which is zwitterionic and has a significantly smaller headgroup compared to ganglioside and PEG-ylated lipids.

**Fig. 4:**
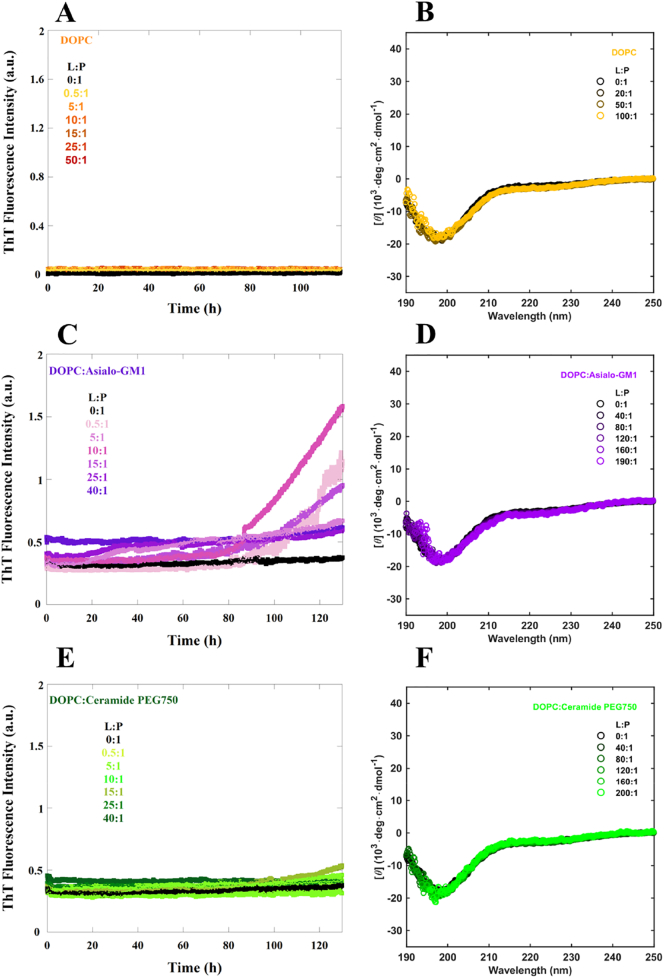
α-Syn aggregation and CD spectra in the presence of uncharged lipid vesicles. A, C and E) Aggregation kinetics of 20 μM α-syn monomer in the presence of DOPC (orange), 9:1 DOPC:Asialo-GM1 (purple) and 9:1 DOPC:Cer-PEG750 (green) monitored using ThT fluorescence under quiescent conditions in 10 mM MES pH 5.5 buffer at 37 °C for concentrations ranging from 0.01 to 1 mM, with the lipid-to-protein molar ratios given in each panel. The median experimental replicate for each condition is shown as a solid line. B, D and F) Far UV CD spectra of 5 μM α-syn alone and in the presence of DOPC (orange), 9:1 DOPC:Asialo-GM1 (purple) and 9:1 DOPC:Cer-PEG750 (green) at concentrations ranging from 0.2 to 1 mM with the lipid-to-protein molar ratios given in each panel.

When monitoring the aggregation kinetics and conformational change for the uncharged systems different behaviors were observed (Fig. 4). In the presence of pure DOPC lipid membranes, no aggregation and no conformational change of α-syn were detected (Fig. 4A-B). In the presence of either Asialo-GM1 or Cer-PEG750, both with large hydrophilic headgroups lacking negative charge, there was also no increase in α-helical CD signal, implying no conformational change of α-syn (Fig. 4D, F). Intriguingly, both Asialo-GM1 and Cer-PEG750 trigger aggregation (Fig. 4C,E), although with much less efficiency than ganglioside, PE-PEG750 and DOPS containing vesicles. This implies that lipids that contain large hydrophilic headgroups may trigger aggregation even in the absence of negative charge, although the accelerating effect is much stronger for the anionic lipids.

### 2.4 Influence of sugars on α-syn aggregation

**Fig. 5:**
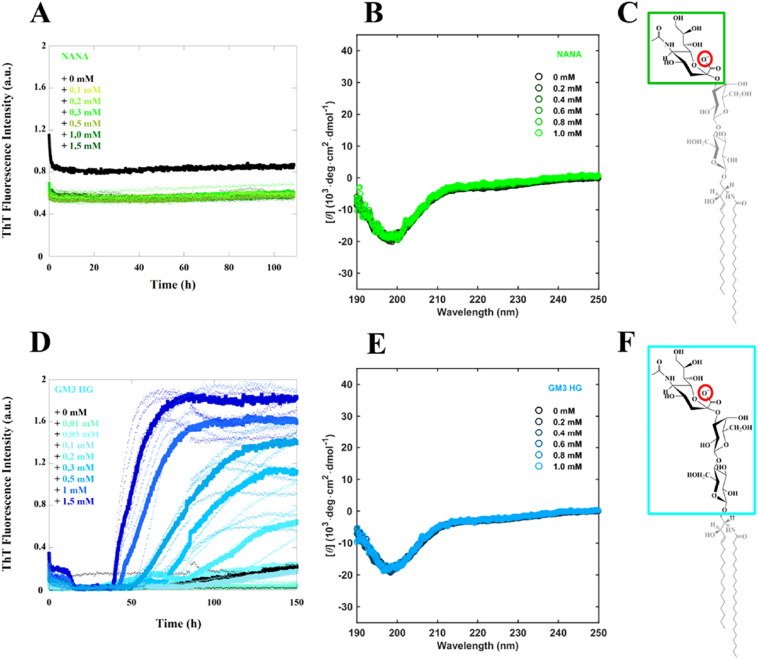
α-Syn aggregation and CD spectra in the presence of NANA and GM3 headgroup molecules. A and D) Aggregation kinetics of 20 μM α-syn monomer in the presence of a concentration variation of NANA or GM3 headgroup molecules in 10 mM MES pH 5.5 buffer under quiescent conditions at 37 °C monitored by ThT fluorescence. The median experimental replicate for each condition is shown as solid lines with other experimental replicates dotted below. B and E) Far UV CD spectra of 5 μM α-syn alone and in the presence of NANA or GM3 headgroup molecules at different ratios. C and F) Chemical structures of NANA and GM3 headgroup molecules and how they relate to the full GM3 lipid molecule.

In a next step, we looked for molecular specificity in α-syn aggregation, in particular, focusing on the headgroup components of the lipid systems studied above. When α-syn was incubated together with negatively charged GM3 headgroups, aggregation of the protein was observed, however, CD spectra of α-syn monomers revealed no conformational change (Fig. 5D-F). Even after 24 h of incubation of α-syn with GM3 headgroups, no changes in secondary structure were observed (Fig. SI1A). Furthermore, ^1^H–^15^N-HSQC NMR spectra showed no effects of adding free GM3 headgroups on the chemical shifts and NMR intensities of α-syn, thus no direct strong interactions could be detected (Fig. SI1B and C). These results altogether suggest that large negatively charged hydrophilic molecules can trigger α-syn aggregation in the absence of any strong association.

**Fig. 6:**
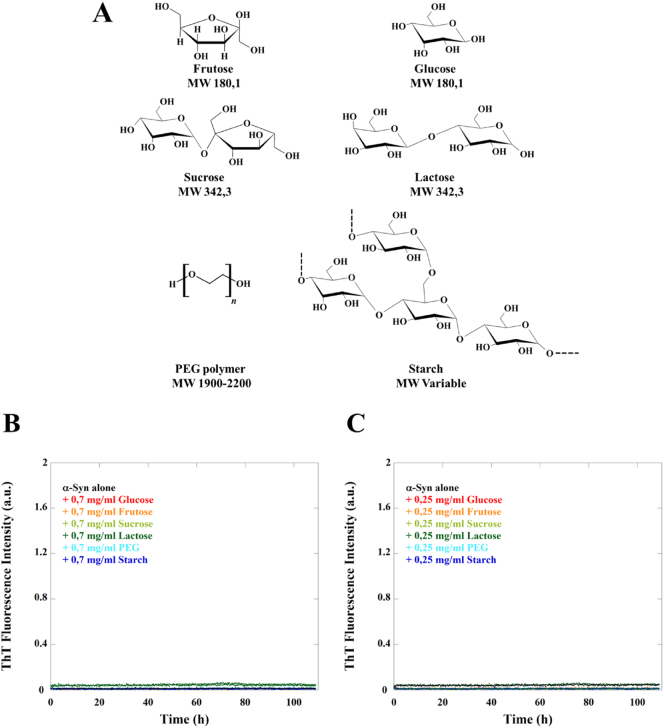
α-Syn aggregation in the presence of different molecules. A) Chemical structures of the molecules investigated. Aggregation kinetics of 20 μM α-syn monomer in the presence of (B) 0,7 mg/ml and (C) 0,25 mg/ml of different sugar and PEG solutions in 10 mM MES pH 5.5 under quiescent conditions at 37 °C monitored by ThT fluorescence. The median experimental replicate for each condition is shown as a solid line with other experimental replicates dotted.

In order to further dissect the effect of free GM3 headgroup, we incubated α-syn with NANA, a small negatively charged sugar residue, representing the charged part of GM3. In this case, no aggregation was observed, and similar to the free GM3 headgroup, the protein remains unstructured (Fig. 5A-C). We also investigated the effects of a range of other hydrophilic molecules, including uncharged sugars varying in size, as well as, PEG of molecular weight ranging from 1900 to 2200 (Fig. 6A). Incubating α-syn with monosaccharides (glucose and fructose), disaccharides (sucrose and lactose) and polymers (starch and PEG), revealed no effects on aggregation during the time frame of the experiment (Fig. 6B-C).

## 3 Discussion

The binding of α-syn to cell membranes has been associated with both its physiological and pathological roles [53, 58, 59]. *In vivo*, α-syn has been shown to partition between free cytosolic and membrane-associated forms [60]. *In vitro* studies have shown that the interaction between α-syn and lipid membranes strongly depends on the lipid composition, and in particular the presence of anionic lipids has shown to be crucial for membrane association. Numerous studies have reported that α-syn associates preferentially to membranes that contain phospholipids with acidic headgroups, for example, PS, PG and PA [9, 20, 23, 52, 61]. Gangliosides constitute another class of anionic lipids, which differ from the anionic phospholipids in that they contain large oligosaccharide headgroups. The difference in headgroup chemistry may influence the location of the protein at the membrane interface, including its penetration depth. Furthermore, gangliosides can be heterogeneously distributed in phospholipid bilayers [78], and protein adsorption and nucleation may occur in ganglioside-containing nanodomains or at defects at domain boundaries, as previously suggested for Aβ [78]. Heterogenous distribution of lipids in the bilayer may also occur for the PEG-ylated lipids as well as the uncharged ganglioside lipids. It is also possible that there are specific interactions between α-syn and the sugar moiety in the lipid headgroups.

The observed binding of α-syn to negatively charged vesicles is attributable to the strong polarity of the net negative α-syn. The accumulation of negative charges in the C-terminus allows this part of the protein to extend from the vesicle surface, thus causing minimum repulsion in the bound state [44]. This enables the rest of the protein to form an amphipathic α-helix embedded in the headgroup layer of the vesicles [9, 48]. The α-helical part contains multiple lysine residues found in the N-terminal region [9, 55], with 7 imperfect 11-residue repeats of KTKEGV sequence (58), similar to those found in apolipoproteins [6, 8].

The CD data in Fig. 3 imply that the helicity increase with increasing lipid concentration (Fig. 3). Still, the slightly less pronounced effect observable for PE-PEG750 compared to GM1, GM3 and DOPS, may be related to that the PEG headgroup contains a more flexible long linear chain, that is different from the bulky branched sugar chains in the ganglioside lipids. These differences may have impact on entropic repulsive interactions (reduced number of configurations of the headgroup after protein binding). Another possible explanation for the observed differences is related to the position of the negative charge within the hydrophilic headgroup. The charge in the ganglioside headgroups is located relatively far out in the headgroup layer (outer surface) and is thus highly accessible from the aqueous solution. In the PEG-ylated lipid, on the other hand, the charge is positioned in between the PEG chain and the acyl-chains, making it more buried into the headgroup layer, and thereby less accessible from the solution. For the reference system containing DOPS, the negative charge is also rather accessible from solution due to the small size of the headgroup. Altogether, the accessibility to the charged group can affect both the attractive interaction between the membrane and α-syn, and the position of the bound α-syn in the hydrophilic headgroup layer. The adsorption of protein may also induce lateral segregation of the lipid species and local change in membrane curvature [[80], [81], [82]].

**Fig. 7:**
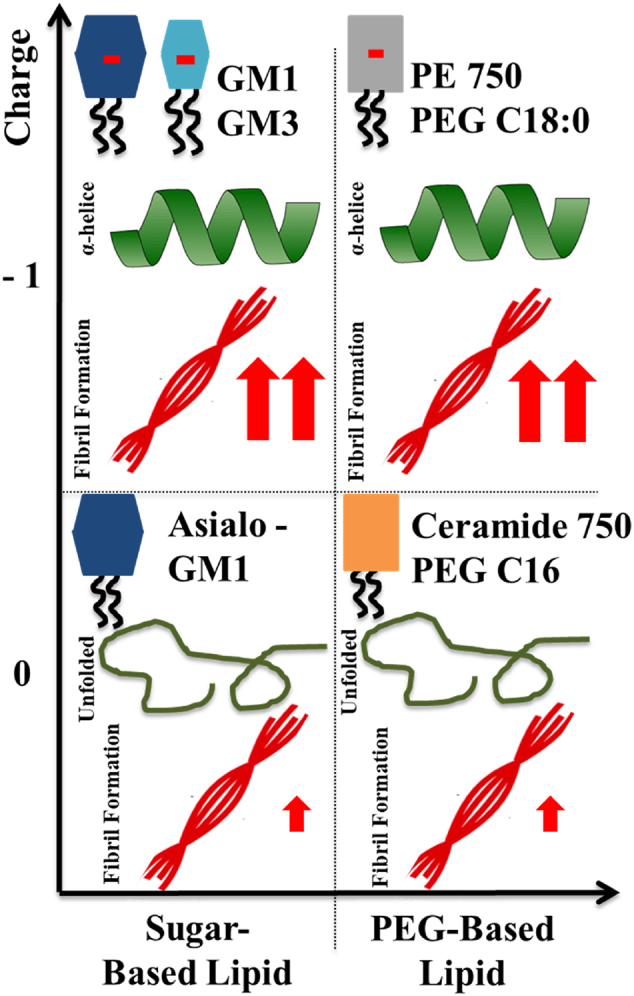
Summary of the results obtained for the aggregation kinetics and conformational change of α-syn in the presence of model membrane systems with different lipid compositions.

The association of α-syn to anionic lipid membranes triggers aggregation by enhancing heterogeneous primary nucleation; this generates nuclei which grow to fibrils, which in turn seed the aggregation reaction with the formation of β-sheet-rich amyloid fibrils. We show that vesicles containing 10 mol% anionic lipids can trigger α-syn aggregation at mildly acidic pH conditions. In an attempt to understand the molecular requirements for the catalytic effect, we compared the ganglioside lipids GM1 and GM3 to other anionic lipids (Fig. 1). PE-PEG750 has a large hydrophilic headgroup of similar size as GM1. It clearly triggers α-syn aggregation, albeit to slightly lower extent than gangliosides. The control model system containing DOPS was also shown to catalyse aggregation. From the combined data in Fig. 2, Fig. 3, we thus conclude that at mildly acidic pH, anionic lipids with both small and large headgroups induce helical conformation of bound α-syn and trigger α-syn aggregation (Fig. 7). The present findings that ganglioside accelerate the aggregation correlate well will previous studies performed at pH 5.5 and higher salt concentrations, showing that ganglioside-containing exosomes purified from N2a cells as well as ganglioside-containing model membranes catalyse α-syn aggregation [21]. The opposite effect has been reported in studies conducted at neutral pH, where GM1 containing vesicles were found to inhibit aggregation [67]. The observed difference between these studies may be a consequence of the differences in pH, lipid phase behavior or the experimental setup.

The combination of data presented here strongly implies that negative charge is more important than the detailed headgroup chemistry, although ganglioside lipids seem to be slightly more catalytic at higher lipid concentrations. The finding that a range of different lipid systems with relatively low content of anionic lipids trigger the aggregation process is clearly different from observations from systematic studies at neutral pH, where only certain lipid systems, including lipids with short (C12-C14) saturated PS will trigger the α-syn aggregation [24]. The mildly acidic pH is relevant to the conditions found in the lumen of endosomes and lysosomes. Locally reduced pH also occurs in mitochondria as a result of oxidative or metabolic stress [79], conditions relevant for PD.

**Fig. 8:**
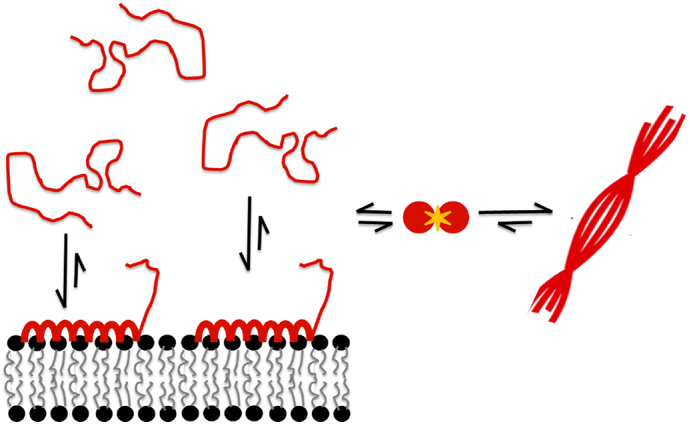
Possible scenario for membrane-induced nucleation of α-syn in the presence of anionic vesicles. Heterogeneous nucleation may be taking place in connection with the exchange process between bound α-helical monomers and unbound relatively unstructured monomers. The formation of nuclei likely involves lipid extraction form the lipid membranes. Also, the formation of nuclei from α-syn intermediates is the rate-limiting step for fibril formation.

An interesting discovery was made when investigating the effect of lipid vesicles that contain only uncharged lipids with small and large headgroups, DOPC, Asialo-GM1 and Cer-PEG750. In the presence of all three lipid systems, α-syn remained unstructured with no α-helical structure detected suggesting too weak interaction to be detected by this method, or a different mode of interaction. For DOPC with a small headgroup, no aggregation was observed. However, both Asialo-GM1 and Cer-PEG750 triggered aggregation to some extent (Fig. 7). Most importantly, these findings suggest that in addition to the negative charge, also charge-independent interactions are important in membrane-induced heterogeneous primary nucleation. We therefore raise the question whether the α-helical state observed in some of the vesicle systems above is on or off pathway towards aggregation. We can rule out being on pathway in the sense of an obligatory conformational detour *via* an α-helical conformation. However, it is possible that nucleation happens in contact with other monomers adsorbed in helical conformation. Thus heterogeneous nucleation may involve the free α-synuclein in transient interactions with adsorbed α-synuclein and the headgroup chemistry may modulate this interaction (Fig. 8). It is also possible that the nucleation takes place in connection with the exchange process between bound α-helical monomers and unbound relatively unstructured monomers. These scenarios are compatible with the observation of the need for excess monomeric protein [20].

**Fig. 9:**
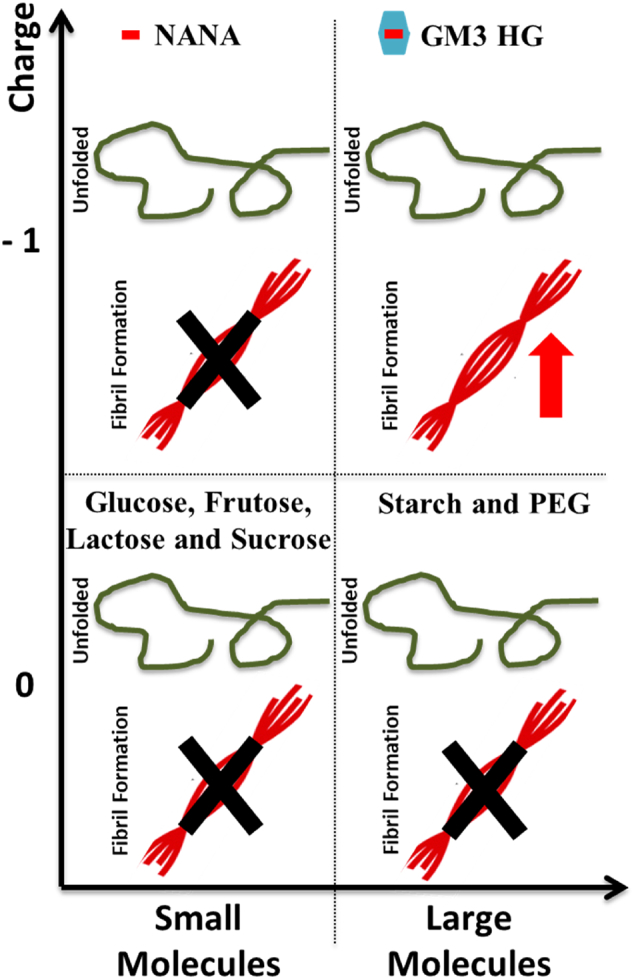
Summary of the results obtained for the aggregation kinetics and conformational change of α-syn in the presence of different molecules varying in size and charge.

Finally, the effect of free ganglioside headgroup and headgroup components were evaluated. In the presence of uncharged molecules of varied sizes (Fig. 6A) no conformational change and no aggregation of α-syn was detected. Intriguingly, aggregation is triggered by free GM3 headgroups while there was no conformational change of α-syn monomers. On the other hand, NANA, the sugar residue carrying the negative charge of ganglioside lipids and of GM3 headgroup, is unable to catalyse α-syn aggregation. Taken together, in the case of free sugars both charge and size of the sugar moiety seem to be important for the interactions with α-syn (Fig. 9). Previous claims that α-syn fibril formation is dependent on the transition of the natively unfolded protein into the aggregation-competent partially folded intermediate [68] are opposed by these findings, which reveal that α-syn helical intermediates are not a required species in triggering of α-syn aggregation.

There is extensive literature suggesting a potential role of ganglioside lipids in PD. Lipidomics studies have found higher plasma levels of GM3 in patients with PD [62]. It has also been suggested that GM1 treatment may have a beneficial regenerative effect on dopaminergic neurons [63]. Understanding the interaction between gangliosides lipids and α-syn is therefore highly relevant. Association between ganglioside lipids, PD and Gaucher disease has been demonstrated [69, 70]. Gaucher disease is the most common lysosomal storage disease and is caused by deficiency of the lysosomal enzyme glucocerebrosidase, resulting in accumulation of glycolipids. Another lysosomal storage disease, Sandhoff disease, results from impaired degradation of GM2, has been shown to lead to accumulation of both α- and β-syn in mouse brains [71]. Interaction with ganglioside lipids has also been observed for another neurodegenerative disease-associated protein, Aβ peptide [72]. Elevated ganglioside concentration is found both in brain and cerebrospinal fluid of AD patients [36, 73]. Ganglioside-bound Aβ identified in cerebrospinal fluid is believed to be involved in seeding of amyloid fibrils in AD [36, 74]. It is further noted that gangliosides are present in exosomes, which are extracellular cell-derived vesicles [21, 75]. Exosomes have during the last years been extensively studied and have been suggested to be a main player in the initiation of the prion-like infectivity of several neurodegenerative diseases. This proposed role of exosomes relies on the ability of exosomes to carry proteins - for example proteins related to neurological diseases, such as PD and AD - and transport them between neighboring cells [75]. Both α-syn and Aβ peptides have been suggested to bind to and cluster with glycosphingolipids, such as GM1, that are particularly concentrated in exosomes [21, 75].

## 4 Concluding remark

The present results have the potential to add to the understanding of the molecular mechanisms involved in amyloid formation in PD. After adipose tissue, neural tissue has the highest content in lipids. Furthermore, in amyloid plaques found in neurons, tightly associated lipids have been identified [10]. The main findings here reported are as follows:

- Lipid membrane composition modulates lipid-protein interactions and fibril formation, and both negative charge and detailed head-group chemistry are important;
- Anionic lipid species with small or large headgroups induce conformational changes of α-syn monomers and catalyse their aggregation;
- Lipid species that have large hydrophilic headgroups and no negative charge trigger α-syn aggregation without conformational change of α-syn monomers. The accelerating effect is, however, far less efficient as compared to the charged lipid systems.
- GM3 headgroup molecules trigger α-syn aggregation in the absence of any strong association, and the effect can be related both to the negative charge and the sugar moiety;
- α-helical α-syn conformation change is not required for lipid-induced aggregation.

## 5 Material and methods

### 5.1 Small unilamelar vesicle preparation

Lipids were obtained lyophilized from Avanti Polar Lipids (Alabaster, AL): 1,2-dioleoyly-*sn*-glycero-3-phosphocholine (DOPC), 1,2-dioleoyl-sn-glycero-3-phospho-l-serine (DOPS), GM1 ganglioside sodium salt from ovine brain (GM1), GM3 ganglioside ammonium salt from milk (GM3), 1,2-distearoyl-*sn*-glycero-3-phosphoethanolamine-N-[methoxy(polyethylene glycol)-750] (PE-PEG750) and N-palmitoyl-sphingosine-1-{succinyl[methoxy(polyethylene glycol)750]} (Cer PEG750). Asialo-Ganglioside GM1 from bovine brain (Asialo-GM1) was obtained from Sigma-Aldrich. To create SUVs of the different lipid systems used for this study a simple and reproducible method was used. Mixtures of these lipids were dissolved in chloroform:methanol 2:1 (v:v). The solvent was then removed by air-drying, creating a thin lipid film deposited onto the glass vial. To certify the complete removal of solvent, the thin lipid film was further dried in a vacuum oven overnight. The hydration of the thin lipid film was done with the desired experimental buffer and vortexed until the solutions appeared milky. The lipid dispersions were then sonicated using a tip sonicator for 15 min using a pulse sequence (10 s on/off duty and 65% amplitude). The clear lipid dispersions were then centrifuged for 5 min at 13000 rpm in order to remove any metallic particles from the sonicator probe.

### 5.2 Protein sample preparation

Human α-syn was expressed in *Escherichia coli* from a Pet3a plasmid containing a synthetic gene with *E. coli* optimised codons (synthesis and cloning purchased from Genscript, Piscataway, New Jersey). Cultures were grown at 37 °C under 125 rpm shaking conditions in baffled flasks (500 ml LB medium per 2500 ml flask) with growth monitored by measuring OD600. When OD600 was between 0.6 and 1.0, protein expression was induced by adding 0.4 mM IPTG with growth continued for another 4 h. Harvesting of the cells was done by centrifugation (10 min at 6000 *g*). α-Syn was purified using heat treatment, ion exchange and gel filtration chromatography, as previously described [51]. For every experiment, α-syn was purified by size exclusion (Superdex 75, GE Healthcare) in the desired experimental buffer and kept on ice. The central fraction of the monomer peak was collected and the concentration of the peptide was determined by absorbance at 280 nm using an extinction coefficient ε = 5800 l.mol^−1^ cm^−1^.

#### 5.2.1 Thioflavin-T kinetic aggregation assay

To monitor the fibril formation process, 100 μl samples were aliquoted in 96 well non-binding PEG-ylated plates (Half-area, 3881 Corning plates), supplemented with 20 μM of ThT and sealed with a plastic film to avoid evaporation. Plates were incubated at 37 °C in a plate reader (FluoStar Omega or FluoStar Galaxy, BMG Labtech, Offenburg, Germany) under quiescent conditions. ThT fluorescence intensity was measured through the bottom of the plate with excitation filter 440 nm and emission filter 480 nm.

### 5.3 Circular dichroism spectroscopy

Circular dichroism spectra were recorded using a JASCO J-715 spectropolarimeter with a PTC-348WI peltier type temperature control system. Samples were prepared by mixing α-syn and the desired lipid or lipid headgroup components followed by 15 min of equilibration at the measurement temperature. The samples were measured in a 1 mm path length quartz cuvette (Hellma 110-QS). 10 mM MES pH 5.5 buffer with the lipid or lipid headgroup component were used as blanks and their spectra subtracted from the spectra of the samples before plotting.

### 5.4 NMR spectroscopy

^1^H–^15^N-HSQC spectra were recorded for samples of 20 μM ^15^N-labeled α-syn in 10 mM MES buffer pH 5.5 at 25 °C at a static magnetic field of 14.1 T using 48 scans, an inter-scan-delay of 1 s, and 128 complex data points in the indirect dimension. Experiments were conducted on free α-syn and α-syn in the presence of 0.5 mM GM3 headgroup molecules. Spectra were processed with NMRPipe [76] and analyzed with NMRView [77].

## Appendix A Supplementary data

Supplementary material Image 1
