# Supplementary material for: Ganglioside lipids accelerate α-synuclein amyloid formation
Source: Biochim Biophys Acta. 2018 Oct;1866(10):1062–72. doi: 10.1016/j.bbapap.2018.07.004 (PMC6121081; doi:10.1016/j.bbapap.2018.07.004)
Supplement: Supplementary file 1 — Supplementary material [file mmc1.docx]

**Supporting Information**

**
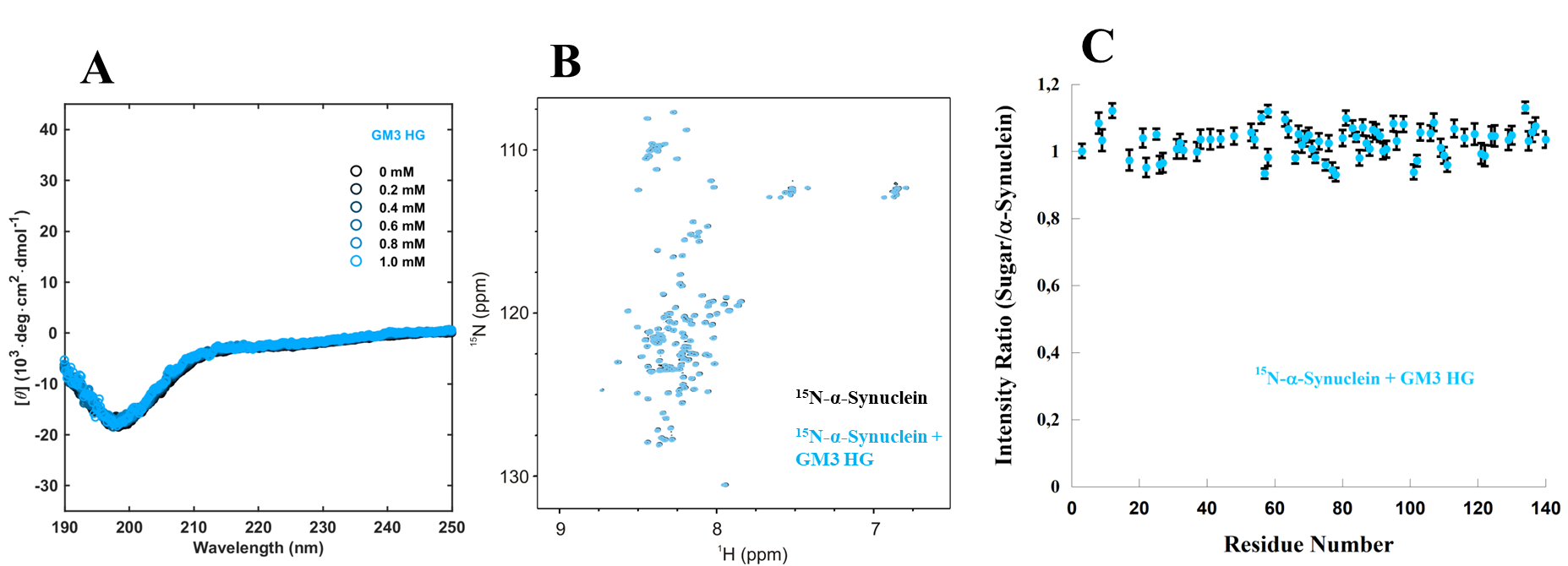
**

**Figure SI1.** α-Syn interaction with GM3 headgroup molecules. **A)** Far UV CD of 5 µM α-syn alone and in the presence of GM3 headgroup molecules incubated for 24 h at different lipid:protein ratios. **B)** Overlay of ^1^H-^15^N-HSQC spectra of 20 µM ^15^N-α-syn alone (black) and in the presence of 0.5 mM GM3 headgroup molecules (light blue). **C)** Relative intensities derived from ^1^H-^15^N-HSQC spectra of 20 µM ^15^N-α-syn in the absence and presence of 0.5 mM GM3 headgroup molecules. The intensity ratios (with/without) for each residue obtained from the ^1^H-^15^N-HSQC spectra is plotted (light blue).
